# Comorbid conditions and the transition among states of hip osteoarthritis and symptoms in a community-based study: a multi-state time-to-event model approach

**DOI:** 10.1186/s13075-020-2101-x

**Published:** 2020-01-20

**Authors:** Carolina Alvarez, Rebecca J. Cleveland, Todd A. Schwartz, Jordan B. Renner, Louise B. Murphy, Joanne M. Jordan, Leigh F. Callahan, Yvonne M. Golightly, Amanda E. Nelson

**Affiliations:** 1 Thurston Arthritis Research Center, University of North Carolina at Chapel Hill, 3300 Doc J. Thurston Building, Campus Box #7280, Chapel Hill, NC 27599-7280 USA; 2 Department of Biostatistics, Gillings School of Global Public Health, University of North Carolina at Chapel Hill, 3106E McGavran-Greenberg Hall, Campus Box #7420, Chapel Hill, NC 27599-7420 USA; 3 Department of Radiology, University of North Carolina at Chapel Hill, 509 Old Infirmary Bldg, Campus Box #7510, Chapel Hill, NC 27599-7510 USA; 4 Centers for Disease Control and Prevention, 4770 Buford Highway NE MS S106-7, Atlanta, GA 30341 USA; 5 School of Medicine, University of North Carolina at Chapel Hill, Chapel Hill, USA; 6 Department of Epidemiology, Gillings School of Global Public Health, University of North Carolina at Chapel Hill, Chapel Hill, USA; 7 Injury Prevention Research Center, University of North Carolina at Chapel Hill, Chapel Hill, USA; 8 Division of Physical Therapy, Department of Allied Health, University of North Carolina at Chapel Hill, Chapel Hill, USA

**Keywords:** Hip osteoarthritis, Comorbidities, Race and gender differences

## Abstract

**Background:**

We examined the association of three common chronic conditions (obesity, diabetes mellitus [DM], and cardiovascular disease [CVD]) with transitions among states of hip osteoarthritis (HOA).

**Methods:**

This longitudinal analysis used data from the Johnston County Osteoarthritis Project (JoCo OA, *n* = 3857), a community-based study in North Carolina, USA, with 18.4 ± 1.5 years of follow-up. Transitions across the following states were modeled: development of radiographic HOA (rHOA; Kellgren-Lawrence grade [KLG] of< 2); development of hip symptoms (self-reported hip pain, aching, or stiffness on most days) or symptomatic HOA (sxHOA; rHOA and symptoms in the same hip), and resolution of symptoms. Obesity (body mass index ≥ 30 kg/m^2^) and self-reported DM and CVD were the time-dependent comorbid conditions of interest. Markov multi-state models were used to estimate adjusted hazard ratios and 95% confidence intervals to describe the associations between the conditions and HOA states.

**Results:**

The sample included 33% African Americans, 39% men, with a mean (SD) age of 62.2 (9.8) years; the frequencies of the comorbidities increased substantially over time. When considered individually, obesity was associated with incident hip symptoms, while CVD and DM were associated with reduced symptom resolution. For those with > 1 comorbidity, the likelihood of incident sxHOA increased, while that of symptom resolution significantly decreased. When stratified by sex, the association between obesity and incident symptoms was only seen in women; among men with DM versus men without, there was a significant (~ 75%) reduction in symptom resolution in those with rHOA. When stratified by race, African Americans with DM, versus those without, were much more likely to develop sxHOA.

**Conclusions:**

Comorbid chronic conditions are common in individuals with OA, and these conditions have a significant impact on the persistence and progression of HOA. OA management decisions, both pharmacologic and non-pharmacologic, should include considerations of the inter-relationships between OA and common comorbidities such as DM and CVD.

## Background

Osteoarthritis (OA) in general has been associated with a substantially higher risk of cardiovascular disease (CVD) [1] and of premature mortality [2], although some of this increased risk is likely explained by walking disability [3]. Conditions such as CVD and diabetes mellitus (DM) are commonly comorbid with OA and have been associated with poorer outcomes, for example following joint replacement [4]. Hip OA (HOA) is a common chronic condition, which will affect a quarter of the population by age 85 [5]. The evidence that OA is associated with CVD, DM, or features of the metabolic syndrome (e.g., hyperglycemia, insulin resistance, obesity, and dyslipidemia) is mixed and is overall stronger for knee OA compared with HOA [6]. The evidence of a specific association between HOA and CVD remains undecided [7], with few studies focused on DM.

Similarly, while obesity is a clear and well-known risk factor for knee OA, its relationship to HOA is less established. The majority of cohort studies to date have found strong associations between obesity and knee OA but no or modest ones for HOA [8]. A 2011 systematic review of 14 studies reported a significant but modest positive association between body mass index (BMI) and HOA, where HOA risk rose by 10% with each increasing unit (kg/m^2^) of BMI (risk ratio of 1.10 [95% confidence interval 1.07–1.16]) [9]. However, most previous studies were cross-sectional, limiting the ability to determine cause and effect. The Johnston County OA Project has extensive longitudinal data on HOA, including symptoms and radiographs and the presence and development of obesity and comorbid conditions, including DM and CVD. Using this unique dataset, we aimed to determine the associations between prevalent or incident obesity, DM, and CVD and the transitions among key states of HOA (e.g., development or resolution of symptoms, or development of radiographic damage).

## Patients and methods

### Study participants

**Fig. 1:**
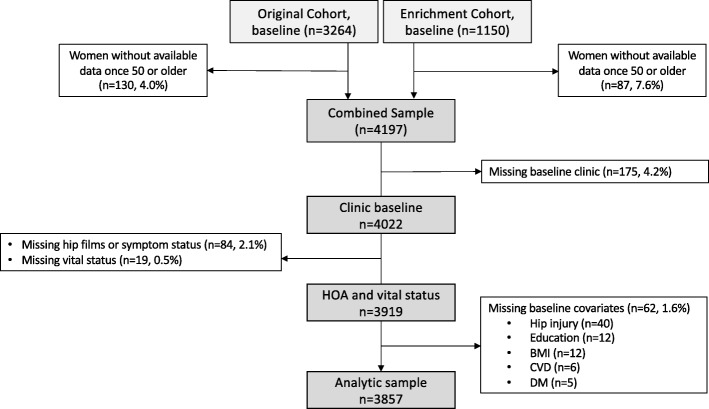
Flowchart of participant inclusion/exclusion at baseline

The study sample, drawn from a community-based, prospective observational cohort of civilian, noninstitutionalized African American and white men and women in Johnston County, North Carolina, USA, consisted of an original cohort (baseline data collection 1991–1997) and enrichment cohort (baseline data collection 2003–2004, enrolled to replace losses from the original cohort over time), as previously described [10]; this study has been approved by the University of North Carolina (IRB 92-0583). All participants were at least 45 years of age at enrollment, although women under 50 years of age did not undergo pelvis radiography per protocol; pelvis radiography for women was added at the visit where they were 50 years of age or older (which was considered their baseline visit). Follow-up data were collected during 1999–2003 for the original cohort, and 2006–2011 and 2013–2015 for both the original and enrichment cohorts (Fig. 1). Vital status for all participants was assessed through the National Death Index up to December 31, 2015. From the initial study sample from both cohorts of 3919 participants with hip X-rays and mortality data, less than 2% (*n* = 62) were missing at least one baseline covariate and were excluded. A complete case analysis was conducted on the remaining 3857 individuals in the analytic sample. Of the additional participants who were lost to follow-up, about 2/5 were due to lack of interest, with the remainder evenly split among the following: moving out of the study area, being physically/mentally unable to participate, or inability to contact. These participants were generally younger, less educated, and more often from the enrichment cohort. A sensitivity analysis limited to those with at least two follow-up time points was performed to assess the impact of loss to follow-up.

### Outcomes: rHOA and symptom assessment

**Fig. 2:**
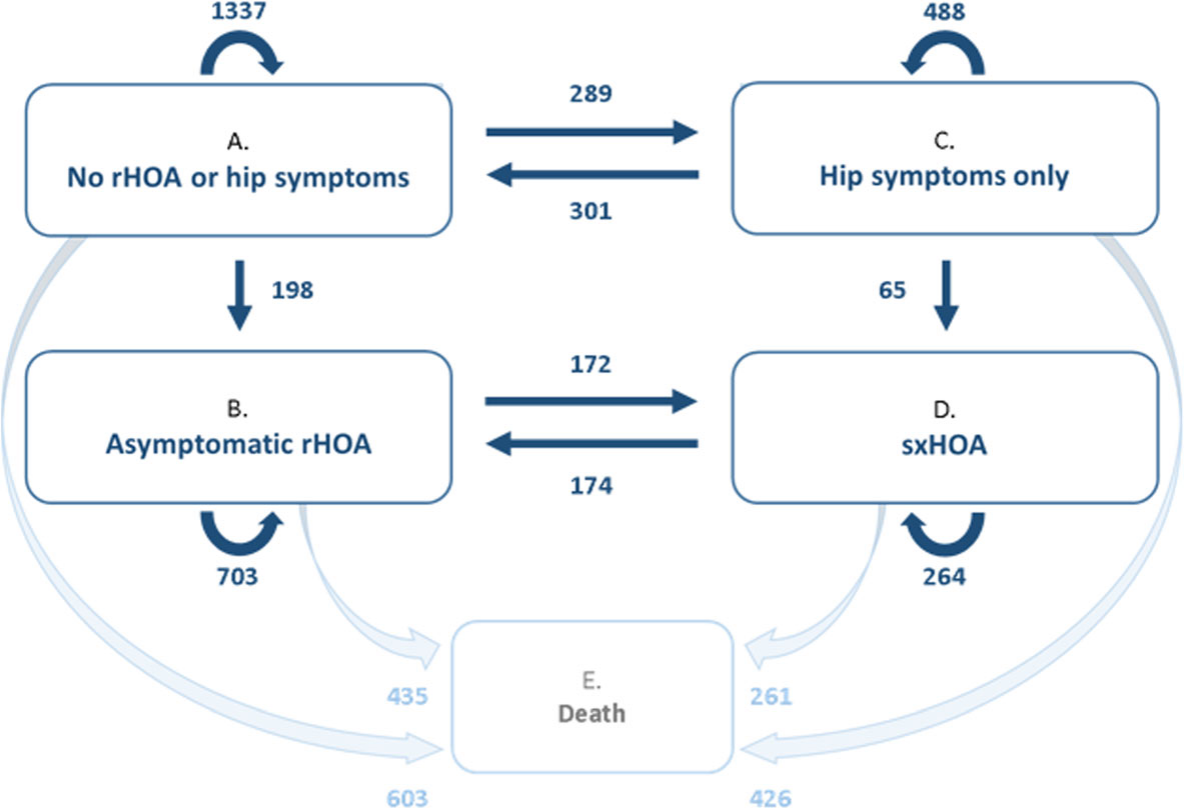
Five-state progressive model for hip status. The number of times each pair of states was observed at successive follow-up times is indicated next to its respective transition arrow. Numbers indicate number of transition instances, not individuals, over the full follow-up period. Diagonal states, while possible, were infrequent and were excluded for parsimony

Participants were classified as having the following outcomes of interest, if present in at least one hip: (1) radiographic HOA (rHOA) as Kellgren-Lawrence grade ≥ 2; (2) hip symptoms defined by self-reported hip pain, aching, or stiffness on most days; (3) symptomatic hip osteoarthritis (sxHOA) defined by both rHOA and symptoms in the same hip. In cases where the hips were disparate within a person, rHOA status was considered first (e.g., if a participant had one hip with symptoms but no rHOA and one hip with asymptomatic rHOA, that person was classified as asymptomatic rHOA). The states of transition modeled were (1) neither rHOA nor hip symptoms (state A); (2) asymptomatic rHOA (rHOA without symptoms, state B); (3) hip symptoms only (symptoms without rHOA, state C); (4) sxHOA (state D); (5) death (state E) as an absorbing state, i.e., a state that cannot be left once entered (Fig. 2). Hip replacements were infrequent in this cohort (at baseline, 15 participants with at least one THR; subsequent incident THRs in 17, 37, and 21 participants by the first, second, and third follow-ups, respectively, for a total of 90) and were included in the analysis as having either rHOA (if no symptoms present) or sxHOA (if symptoms were present).

### Main effects: comorbid conditions

The three comorbidities were defined separately at each study visit. Obesity was defined as a measured BMI of at least 30.0 kg/m^2^. For self-reported conditions, participants were read the following statement: “Please tell me which of the following conditions or illnesses a doctor, nurse, or health professional has told you that you have now or have ever had.” Self-reported DM status was elicited through a yes/no answer to “diabetes or high blood sugar.” Across data collection cycles, CVD status was assessed with increasing inclusiveness and specificity (baseline: heart attack, other heart problems, cerebrovascular accident; first follow-up: added angina, congestive heart failure; second follow-up: added peripheral vascular disease). All comorbidities were assessed as time-dependent, meaning that their presence could change across time for a given participant; for obesity, both development and resolution were possible, but DM and CVD could only develop and not resolve.

### Statistical analysis

To meet this study’s objectives, we chose an advanced method to allow modeling of several transitions of interest, which incorporated varying or unequally spaced times of transition, and included covariates which changed over time. The models include transitions across multiple events including condition worsening and improvement. A time-to-event analysis was performed using data from baseline and up to three follow-up time points. Markov multi-state models (MSM) for interval-censored outcomes (i.e., outcomes occurring during the interval between assessment time points) were conducted using R software and the MSM package. [11] MSM are based on the theory of stochastic processes, which describe a collection of random variables representing the evolution of a process over time. They assess how an individual (rather than a hip) transitions between states in continuous time under the Markov assumption, where future transition depends only on the current state. We used time-inhomogeneous, piecewise exponential models to model and change intensities for all participants at age 65; in other words, the exponential parametric model was assumed constant at two separate intervals (while < 65 or while 65 years or older), recognizing that the standard assumption of constant risk for these transitions does not capture the known effect of increased age. Therefore, in addition to adjusting the model for age, the estimates were allowed to change based on a threshold at age 65; given the clinical and sample-based (near the median) plausibility of this threshold, no other age thresholds were used.

Adjusted hazard ratios (aHRs) and corresponding 95% confidence intervals (95% CI) were estimated to determine independent associations between each comorbidity and each hip state transition, individually and in combination, in a five-state progressive model. Pairwise combination models were used to determine effects when each pair of comorbidities occurred concurrently, compared to the absence of those two comorbidities. A separate combination model was used to determine transition effects when all three comorbidities were present, compared to the absence of all three comorbidities. The transitions modeled using MSM were development of rHOA (either A to B or C to D shown in Fig. 2), development of symptoms (A to C or B to D), or resolution of symptoms (C to A or D to B). While diagonal transitions are also possible, these were infrequent (35 instances of transition from states A to D, 64 transitions from states C to B), indicating a transitional state with the final event being rHOA, and thus were dropped from the model for simplicity. Additionally, transitions to death (the absorbing state) were modeled but results of main effects on these transitions are not shown as this was not the aim of our study. Qualitative model assessment was conducted by visual consideration of observed and expected prevalence plots for each state. We adjusted for covariates assumed to be either static or changing at observed times (if time-dependent). All models were person-based and were adjusted for relevant baseline demographics (age, sex, race, and education [< 12 years]), self-reported, time-dependent history of hip injury or fracture, cohort (original or enrichment), and mean-centered birth year. Stratification by race and sex was performed for each of the individual comorbidities in an exploratory manner given sample size limitations (which did not permit stratified analyses of combinations of comorbidities).

## Results

### Descriptive results

The three follow-up visits took place approximately every 6 years. At baseline, the sample included 33% African Americans, 39% men, with a mean (SD) age of 62.2 (9.8) years, and 37% with less than 12 years of education (Table 1). At that time, 45% had no rHOA or hip symptoms, 25% had hip symptoms only, 19% had asymptomatic rHOA, and 11% had sxHOA. By the end of the follow-up period (i.e., third follow-up), asymptomatic rHOA had increased to 34% and sxHOA had increased to 15%. In other words, of the 1743 participants starting without rHOA or symptoms, 26% (452) went on to transition to states of hip OA, symptoms, or both. The rest (*n* = 1291) did not transition by the third follow-up visit, having died (*n* = 748) or being censored (*n* = 543). Only 6% reported a hip injury or fracture at baseline, which doubled over the follow-up period. The majority of the sample reported no symptoms in their hips at all time points, although all categories from mild to severe were represented (Table 1); women were more likely to report symptoms than men, with no differences by race (data not shown). Forty percent of the baseline sample met criteria for obesity, increasing to nearly 50% by the last follow-up. Similarly, the percentage of both DM and CVD approximately doubled over time (from 14 to 28% for DM and 22 to 48% for CVD), although the cumulative incidence over each time point was relatively stable (9–10% for DM and 11–13% for CVD, Table 1).

**Table 1:** Descriptive characteristics of JoCo OA participants with complete data (*n* = 3857)

	Study visit (mean ± SD years from baseline)							
	Baseline *n* = 3857		1st follow-up *n* = 2300 (6.0 ± 1.2 years)		2nd follow-up *n* = 1336 (12.2 ± 1.5 years)		3rd follow-up *n* = 454 (18.4 ± 1.5 years)	
Characteristics†	*n*	*%*	*n*	*%*	*n*	*%*	*n*	*%*
Age (years, mean ± SD))	62.2	± 9.8	67.0	± 9.2	71.1	± 7.8	74.9	± 6.7
Original cohort (vs enrichment)	2732	70.8	1617	70.3	960	71.9	454	100
Men	1495	38.8	808	35.1	461	34.5	189	41.6
African American	1269	32.9	710	30.9	403	30.2	114	25.1
< 12 years education	1412	36.6	701	30.5	298	22.3	68	15.0
Hip injury	245	6.4	200	8.7	125	9.4	52	11.5
Obesity (BMI ≥ 30 kg/m^2^)	1543	40.0	1061	46.1	663	49.6	222	48.9
Prevalent DM	532	13.8	457	19.9	340	25.4	127	28.0
Incident DM			196	8.5	114	8.5	43	9.5
Prevalent CVD	855	22.2	734	31.9	557	41.7	217	47.8
Incident CVD			302	13.1	159	11.9	50	11.0
Hip symptoms (max)^$^								
None	2403	62.3	1512	65.7	928	69.5	300	66.1
Mild	405	10.5	251	10.9	137	10.3	46	10.1
Moderate	645	16.7	343	14.9	181	13.5	44	9.7
Severe	375	9.7	194	8.4	90	6.7	64	14.1
State definitions								
No rHOA or symptoms	1743	45.2	947	41.2	531	39.7	160	35.2
Asymptomatic rHOA	736	19.1	564	24.5	421	31.5	154	33.9
Hip symptoms only	967	25.1	505	22.0	201	15.0	71	15.6
SxHOA	411	10.7	284	12.3	183	13.7	69	15.2

*BMI* body mass index in kg/m^2^, measured and calculated from height and weight, *DM* diabetes mellitus, and *CVD* cardiovascular disease, are self-reported (see “Main effects: comorbid conditions” for additional detail. *OA* osteoarthritis, *rHOA* radiographic hip OA, *sxHOA* symptomatic hip OA

†*n*(%) unless otherwise noted

^$^*n* = 29 participants are missing at baseline (this was a separate question than the ANY symptoms question used for the state definitions). The maximum severity for the person, considering both hips, is shown

### Associations among HOA state transitions and individual comorbidities

First, we considered the overall effect of each comorbid condition (i.e., obesity, DM, and CVD) at each visit on the transitions across states of HOA at subsequent visits (Table 2). Compared with individuals without obesity, those with obesity had a significant 33% higher hazard of developing symptoms (states A to C) over the full follow-up period. The association for obesity and development of incident sxHOA was also positive although not statistically significant (aHR 1.46, 95% CI [0.91, 2.36]). Compared to those without CVD, those with or who developed CVD were more likely to develop asymptomatic rHOA (A to B) or to develop symptoms (A to C), although neither association was statistically significant. However, among those with symptoms only, those with CVD were significantly less likely to have symptom resolution than those without CVD (C to A); a similar trend was seen for symptom resolution in those with rHOA although not statistically significant. Having or developing DM was not significantly associated with any of the overall transitions, but similar to CVD, symptoms were less likely to resolve among those with DM compared to those without (states C to A: aHR 0.74, 95% CI [0.51, 1.08] and states D to B: aHR 0.64, 95% CI [0.38, 1.08]).

**Table 2:** Adjusted hazard ratios (aHR) and 95% confidence intervals (CI) for comorbid conditions, individually and in combination, on modeled transition states, over the full follow-up period

Type of transition	Individual comorbid conditions			Combinations of comorbid conditions		
	Obesity (vs no obesity)^1^	DM (vs no DM)^1^	CVD (vs no CVD)^1^	Obesity and CVD (vs: no Obesity and no CVD)^2^	Obesity and DM (vs: no Obesity and no DM)^3^	CVD and DM (vs: no CVD and no DM)^4^
	*n* (vs *n*) for transitions			*n* (vs *n*) for transitions		
	aHR (95% CI)			aHR (95% CI)		
Development of rHOA						
No rHOA/symptoms (A) to rHOA (B)	82 (vs 116)	39 (vs 159)	69 (vs 129)	16 (vs 75)	12 (vs 103)	6 (vs 103)
	0.88 (0.65, 1.18)	1.06 (0.68, 1.63)	1.34 (0.97, 1.85)	1.27 (0.78, 2.06)	0.89 (0.51, 1.55)	0.79 (0.34, 1.85)
Symptoms only (C) to sxHOA (D)	38 (vs 27)	18 (vs 47)	41 (vs 24)	7 (vs 11)	10 (vs 24)	7 (vs 20)
	1.46 (0.91, 2.36)	1.49 (0.86, 2.59)	1.07 (0.65, 1.78)	1.41 (0.63, 3.12)	*2.38 (1.23, 4.63)*	*2.25 (1.08, 4.70)*
Development of symptoms						
No rHOA/symptoms (A) to symptoms only (C)	159 (vs 133)	64 (vs 225)	108 (vs 181)	29 (vs 82)	19 (vs 112)	8 (vs 144)
	*1.33 (1.01, 1.74)*	1.05 (0.71, 1.54)	1.25 (0.92, 1.71)	*1.91 (1.21, 3.01)*	1.36 (0.84, 2.18)	1.01 (0.50, 2.03)
rHOA (B) to sxHOA (D)	87 (vs 85)	39 (vs 133)	71 (vs101)	17 (vs 48)	15 (vs 71)	6 (vs 81)
	0.93 (0.63, 1.35)	1.29 (0.78, 2.15)	1.08 (0.71, 1.63)	0.95 (0.52, 1.73)	0.83 (0.43, 1.61)	0.98 (0.38, 2.51)
Resolution of symptoms						
Symptoms only (C) to no rHOA/symptoms (A)	164 (vs 137)	67 (vs 234)	107 (vs 194)	34 (vs 85)	26 (vs 115)	14 (vs 151)
	0.88 (0.67, 1.14)	0.74 (0.51, 1.08)	*0.61 (0.45, 0.82)*	*0.56 (0.36, 0.86)*	0.65 (0.41, 1.04)	*0.46 (0.25, 0.84)*
sxHOA (D) to rHOA (B)	104 (vs 70)	38 (vs 136)	72 (vs 102)	23 (vs 41)	10 (vs 58)	8 (vs 80)
	0.85 (0.58, 1.23)	0.64 (0.38, 1.08)	0.74 (0.50, 1.09)	0.62 (0.35, 1.10)	*0.35 (0.17, 0.71)*	*0.44 (0.21, 0.94)*

^1^Model includes effects for baseline values of birth year, study cohort, age, sex, race, and education and time-dependent values of obesity, DM, CVD, and hip injury

^2^Model includes effects for baseline values of birth year, study cohort, age, sex, race, and education and time-dependent values of hip injury, DM, group of obesity with no CVD, group of no obesity with CVD, and group of obesity with CVD

^3^Model includes effects for baseline values of birth year, study cohort, age, sex, race, and education and time-dependent values of hip injury, CVD, group of obesity with no DM, group of no obesity with DM, and group of obesity with DM

^4^Model includes effects for baseline values of birth year, study cohort, age, sex, race, and education and time-dependent values of hip injury, obesity, group of CVD with no DM, group of no CVD with DM, and group of CVD with DM

Italics indicate statistical significance (i.e., the 95% CI excludes 1)

### Associations among HOA state transitions and multiple comorbidities

When the comorbidities were instead considered jointly (Table 2), the combination of obesity and CVD (compared with the absence of both conditions) nearly doubled the rate of symptom worsening (states A to C), while also reducing the rate of symptom resolution by half among those without rHOA (states C to A). Symptom resolution among those with both conditions and rHOA was also reduced but was not statistically significant (states D to B, aHR 0.62, 95% CI [0.35, 1.10]). The combination of obesity and DM, or of CVD and DM, compared with the absence of both conditions, resulted in more than twice the risk of developing rHOA in those with symptoms (incident sxHOA, states C to D) and was statistically significant. These combinations (obesity and DM, or CVD and DM) also resulted in significantly lower risk of symptom resolution among those with or without rHOA (states D to B and C to A, respectively, Table 2). Additionally, when considering all three comorbidities versus none in a combined model, similar patterns were seen, in that individuals with obesity, DM, and CVD compared to those with none of these comorbidities were substantially less likely to experience symptomatic resolution regardless of rHOA status (states C to A, aHR 0.39, 95% CI [0.18, 0.83]; states D to B, aHR 0.22, 95% CI [0.08, 0.60]), and had nearly four times the likelihood of developing incident sxHOA (state C to D, aHR 3.71, 95% CI [1.44, 9.58]; data not shown).

### Exploratory stratified analyses

When stratified by sex, the overall pattern was similar, with a few notable differences (Table 3). Although in the same direction, the association between obesity and development of symptoms (states A to C) was significant in women (aHR 1.44, 95% CI [1.02, 2.02]) but not in men (aHR 1.15, 95% CI [0.74, 1.79]), as was the statistically non-significant but suggestive association between CVD and development of asymptomatic rHOA (states A to B; aHR for women 1.42, 95% CI [0.98, 2.05); for men aHR 1.08, 95% CI [0.53, 2.19)]. Among men only, there was a significant reduction in symptom resolution in the presence of rHOA for those with DM versus those without (states D to B, aHR 0.28, 95% CI [0.10, 0.81]), although the direction of the non-significant associations in women and for other outcomes was generally consistent. Resolution of symptoms (states C to A, or D to B) was less likely in the presence of CVD, but this association was only statistically significant in women.

**Table 3:** Adjusted hazard ratios (aHR) and 95% confidence intervals (CI) for comorbid conditions, individually, on modeled transition states, over the full follow-up period, by sex

Strata	Type of transition	Obesity (vs no obesity)^1^	DM (vs no DM)^1^	CVD (vs no CVD)^1^
		*n* (vs *n*) for transitions		
		aHR (95% CI)		
Women	Development of rHOA			
	No rHOA/symptoms (A) to rHOA (B)	56 (vs 77)	25 (vs 108)	49 (vs 84)
		0.85 (0.59, 1.21)	1.03 (0.61, 1.75)	1.42 (0.98, 2.05)
	Symptoms only (C) to sxHOA (D)	28 (vs 21)	13 (vs 36)	32 (vs 17)
		1.27 (0.73, 2.20)	1.42 (0.74, 2.75)	1.23 (0.70, 2.17)
	Development of symptoms			
	No rHOA/symptoms (A) to symptoms only (C)	108 (vs 77)	39 (vs 146)	64 (vs 121)
		*1.44 (1.02, 2.02)*	0.92 (0.56, 1.50)	1.22 (0.83, 1.79)
	rHOA (B) to sxHOA (D)	55 (vs 59)	26 (vs 88)	51 (vs 63)
		0.70 (0.43, 1.13)	1.42 (0.78, 2.61)	0.94 (0.58, 1.54)
	Resolution of symptoms			
	Symptoms only (C) to no rHOA/symptoms (A)	122 (vs 86)	48 (vs 160)	75 (vs 133)
		0.93 (0.68, 1.27)	0.78 (0.49, 1.22)	*0.60 (0.42, 0.87)*
	sxHOA (D) to rHOA (B)	69 (vs 49)	26 (vs 92)	52 (vs 66)
		0.71 (0.44, 1.13)	0.86 (0.48, 1.54)	*0.60 (0.38, 0.94)*
Men	Development of rHOA			
	No rHOA/symptoms (A) to rHOA (B)	26 (vs 39)	14 (vs 51)	20 (vs 45)
		0.93 (0.55, 1.57)	1.10 (0.52, 2.31)	1.08 (0.53, 2.19)
	Symptoms only (C) to sxHOA (D)	10 (vs 6)	5 (vs 11)	9 (vs 7)
		2.14 (0.88, 5.21)	1.26 (0.46, 3.47)	0.68 (0.21, 2.17)
	Development of symptoms			
	No rHOA/symptoms (A) to symptoms only (C)	48 (vs 56)	25 (vs 79)	44 (vs 60)
		1.15 (0.74, 1.79)	1.32 (0.71, 2.45)	1.26 (0.75, 2.13)
	rHOA (B) to sxHOA (D)	32 (vs 26)	13 (vs 45)	20 (vs 38)
		1.50 (0.78, 2.88)	0.81 (0.33, 2.03)	1.46 (0.65, 3.28)
	Resolution of symptoms			
	Symptoms only (C) to no rHOA/symptoms (A)	42 (vs 51)	19 (vs 74)	32 (vs 61)
		0.78 (0.49, 1.24)	0.70 (0.38, 1.30)	0.61 (0.36, 1.05)
	sxHOA (D) to rHOA (B)	35 (vs 21)	12 (vs 44)	20 (vs 36)
		1.15 (0.60, 2.21)	*0.28 (0.10, 0.81)*	1.12 (0.54, 2.33)

^1^Model includes effects for baseline values of birth year, study cohort, age, race, and education and time-dependent values of obesity, DM, CVD, and hip injury

Italics indicate statistical significance (i.e., the 95% CI excludes 1)

When stratified by race (Table 4), the most striking difference was for African Americans with DM, who, compared to African Americans without DM, had nearly four times the hazard of developing incident symptomatic HOA (states C to D, aHR 3.57, 95% CI [1.10, 11.7]) and had more than twice the hazard of developing symptoms when rHOA was present, although the latter was of borderline statistical significance (states B to D, aHR 2.09, 95% CI [0.97, 4.54]). Additionally, the significant association in the overall analysis between obesity and development of symptoms (states A to C) was seen only in white individuals; the association seen in women between CVD and incident rHOA was also evident only in whites (Table 4).

**Table 4:** Adjusted hazard ratios (aHR) and 95% confidence intervals (CI) for comorbid conditions, individually, on modeled transition states, over the full follow-up period, by race

Strata	Type of transition	Obesity (vs no obesity)^1^	DM (vs no DM)^1^	CVD (vs no CVD)^1^
		*n* (vs *n*) for transitions		
		aHR (95% CI)		
White	Development of rHOA			
	No rHOA/symptoms (A) to rHOA (B)	45 (vs 88)	18 (vs 115)	46 (vs 87)
		0.75 (0.51, 1.11)	0.93 (0.48, 1.80)	1.49 (0.99, 2.24)
	Symptoms only (C) to sxHOA (D)	31 (vs 22)	12 (vs 41)	33 (vs 20)
		1.48 (0.89, 2.44)	1.16 (0.61, 2.23)	1.02 (0.58, 1.77)
	Development of symptoms			
	No rHOA/symptoms (A) to symptoms only (C)	107 (vs 107)	46 (vs 168)	76 (vs 138)
		*1.46 (1.06, 2.00)*	1.20 (0.75, 1.90)	1.31 (0.91, 1.89)
	rHOA (B) to sxHOA (D)	65 (vs 72)	25 (vs 112)	57 (vs 80)
		0.88 (0.65, 1.54)	0.94 (0.47, 1.87)	1.36 (0.84, 2.21)
	Resolution of symptoms			
	Symptoms only (C) to no rHOA/symptoms (A)	100 (vs 99)	38 (vs 161)	69 (vs 130)
		1.03 (0.75, 1.43)	0.68 (0.40, 1.17)	*0.58 (0.39, 0.85)*
	sxHOA (D) to rHOA (B)	73 (vs 59)	29 (vs 103)	55 (vs 77)
		0.87 (0.57, 1.38)	0.58 (0.31, 1.06)	0.87 (0.54, 1.39)
Black	Development of rHOA			
	No rHOA/symptoms (A) to rHOA (B)	37 (vs 28)	21 (vs 44)	23 (vs 42)
		1.11 (0.69, 1.78)	1.10 (0.59, 2.06)	1.17 (0.69, 1.97)
	Symptoms only (C) to sxHOA (D)	7 (vs 5)	6 (vs 6)	8 (vs 4)
		1.09 (0.32, 3.71)	*3.57 (1.10, 11.7)*	1.22 (0.36, 4.04)
	Development of symptoms			
	No rHOA/symptoms (A) to symptoms only (C)	49 (vs 26)	18 (vs 57)	32 (vs 43)
		1.00 (0.59, 1.72)	0.79 (0.40, 1.57)	1.19 (0.66, 2.17)
	rHOA (B) to sxHOA (D)	22 (vs 13)	14 (vs 21)	14 (vs 21)
		0.69 (0.32, 1.46)	2.09 (0.97, 4.54)	0.63 (0.27, 1.49)
	Resolution of symptoms			
	Symptoms only (C) to no rHOA/symptoms (A)	64 (vs 38)	29 (vs 73)	38 (vs 64)
		0.66 (0.42, 1.04)	0.77 (0.46, 1.31)	0.70 (0.43, 1.13)
	sxHOA (D) to rHOA (B)	31 (vs 11)	9 (vs 33)	17 (vs 25)
		0.69 (0.35, 1.39)	0.91 (0.37, 2.24)	0.52 (0.25, 1.09)

^1^Model includes effects for baseline values of birth year, study cohort, age, sex, and education and time-dependent values of obesity, DM, CVD, and hip injury

Italics indicate statistical significance (i.e., the 95% CI excludes 1)

In sensitivity analyses of the results in Tables 2, 3, and 4 limited to those individuals with at least two follow-up time points, magnitudes of effects were not substantially changed (although some were no longer statistically significant due to smaller sample sizes).

## Discussion

This longitudinal analysis using a state transition model identified several associations among common comorbidities and state transitions of HOA in a community-based cohort. Obesity was associated with greater risk of developing symptoms, particularly in women, while CVD and DM reduced the hazard of symptom resolution over time. The effects were stronger for combinations of comorbidities, where most combinations of two comorbidities resulted in a statistically significantly lower hazard of symptom resolution. Additionally, in combination with DM, both obesity and CVD resulted in twice the hazard of incident sxHOA (compared to those without DM or either obesity or CVD). In stratified analyses, African Americans with DM, compared to those without DM, had greater hazard for development of symptoms or sxHOA. These findings reinforce the effects of multiple chronic conditions in individuals with or at risk for HOA.

Higher BMI has been associated with greater self-reported pain and poorer function among individuals awaiting hip replacement surgery, despite similar radiographic severity of disease [12]. Some studies have reported no association between the metabolic syndrome or its individual components in severe HOA [6]. However, one or more comorbid conditions conferred a higher risk of revision of hip arthroplasty (HR 1.16, 95% CI [1.08, 1.23]) in a large Finnish registry; this was mostly attributable to CVD and particularly heart failure [4]. A recent systematic review of comorbidities and the prognosis of clinical symptoms in knee and/or hip OA noted greater pain and poorer performance-based function in those with one or more comorbid conditions; specifically, DM was associated with greater pain, while CVD was more associated with decrements in physical function [13]. This is consistent with our findings of persistent symptoms and greater chance of developing symptomatic OA among those with multiple comorbidities including DM. Another study found a greater likelihood of persistent pain after joint replacement among individuals with DM, but not with metabolic syndrome or obesity [14]. However, in the JoCo OA, the lifetime risk of HOA did not vary substantially by BMI or other demographic features [5].

In exploratory analyses stratified by sex, some of the associations were statistically significant only for women, but this may be due to limitations in sample size for the men, particularly since the effects were generally in the same direction. Interestingly, we did see a significant reduction in symptom resolution in men only for those with DM compared to those without. Because of the smaller numbers, we were not able to consider separately the four race by sex strata, and also we could not assess the effect of combinations of comorbidities in the sex and race strata.

In exploratory analyses stratified by race, we found an unexpected racial difference when modeling DM alone among African Americans: compared with those without DM, individuals with DM were more likely to worsen from hip symptoms only to sxHOA, or from asymptomatic rHOA to sxHOA. This specific finding has not been previously reported. However, there is evidence that, compared with whites, African Americans with OA tend to report greater pain and have poorer self-reported function and greater disability; in some cases, these relationships are partly attenuated by differences in BMI, psychological factors (e.g., depression, pain coping, general health), or occupational exposures [15]. Similarly, compared with whites, African Americans have a greater risk, as well as overall poorer control, of DM and other cardiometabolic conditions (e.g., obesity, hypertension, metabolic syndrome) and are much more likely to develop end-stage renal disease (with or without DM); these disparities are hypothesized to occur partly because of differences in diet and physical activity, socioeconomic status, and access to care [16]. Therefore, this finding may relate to overall poorer management of these comorbid conditions among affected African Americans, who then are more likely to experience progression of HOA, an area deserving of further study.

There are several limitations of this study. Our results in this community-based cohort of individuals in North Carolina may not be representative of other populations of differing ages or race/ethnicity. Additionally, the diagnoses of DM and CVD were based on participant self-report of prior doctor diagnosis rather than direct testing (e.g., we do not have HbA1c values) or medical record review, although self-report of these conditions is fairly reliable [17, 18]. Our data source is an important strength, as it is a racially diverse sample with 18 years of follow-up. Sensitivity analyses indicated no remarkable impact from loss to follow-up. We used an advanced and relatively novel statistical method, which has many benefits, including the ability to model several transitions of interest, rather than focusing on only one or a few outcomes, while still incorporating imprecise times of transition (i.e., interval-censored events, common in cohorts studying chronic disease). Here, we have simultaneously modeled incident rHOA, incident symptoms, incident sxHOA, and resolution of symptoms, allowing inclusion of a larger number of participants over multiple time points. This model also allows for inclusion of time-varying covariates, meaning that we were able to account for changes in obesity status, and new onset or resolution of comorbidities, over time.

## Conclusions

Combinations of common comorbid conditions (i.e., obesity, DM, and CVD) led to higher likelihood of persistence and an increased chance of worsening symptoms or progression to sxHOA, most notably among African Americans with DM. The associations identified in this analysis highlight the combined impact of multiple comorbid conditions including OA and the need to consider multimorbidity in the evaluation and care of these patients.

## Data Availability

The datasets used and/or analyzed during the current study are available from the corresponding author on reasonable request.

## Abbreviations

aHR: Adjusted hazard ratio
BMI: Body mass index
CI: Confidence interval
CVD: Cardiovascular disease
DM: Diabetes mellitus
HOA: Hip osteoarthritis
JoCo OA: Johnston County Osteoarthritis Project
KLG: Kellgren-Lawrence Grade
MSM: Markov multi-state models
OA: Osteoarthritis
rHOA: Radiographic hip osteoarthritis
SD: Standard deviation
sxHOA: Symptomatic hip osteoarthritis
